# Is living in a household with children associated with SARS-CoV-2 seropositivity in adults? Results from the Swiss national seroprevalence study Corona Immunitas

**DOI:** 10.1186/s12916-022-02431-z

**Published:** 2022-06-20

**Authors:** Jacob Blankenberger, Marco Kaufmann, Emiliano Albanese, Rebecca Amati, Daniela Anker, Anne-Linda Camerini, Patricia Chocano-Bedoya, Stéphane Cullati, Alexia Cusini, Jan Fehr, Erika Harju, Philipp Kohler, Susi Kriemler, Gisela Michel, Nicolas Rodondi, Pierre-Yves Rodondi, Alexandre Speierer, Stefano Tancredi, Milo A. Puhan, Christian R. Kahlert, on behalf of the Corona Immunitas Research Group

**Affiliations:** 1 Epidemiology, Biostatistics and Prevention Institute (EBPI), University of Zurich, Zurich, Switzerland; 2 Institute of Public Health, Università della Svizzera Italiana, Lugano, Switzerland; 3 Population Health Laboratory (#PopHealthLab), University of Fribourg, Fribourg, Switzerland; 4 Institute of Primary Health Care (BIHAM), University of Bern, Bern, Switzerland; 5 Population Health Laboratory (#PopHealthLab), University of Fribourg, Fribourg, Switzerland; 6 Department of Readaptation and Geriatrics, Faculty of Medicine, University of Geneva, Geneva, Switzerland; 7 Division of Infectious Diseases, Kantonsspital Graubünden, Chur, Switzerland; 8 Division of Infectious Disease & Hospital Epidemiology, University Hospital Zurich, Zurich, Switzerland; 9 Department of Health Sciences and Medicine, University of Luzern, Luzern, Switzerland; 10 Department of General Internal Medicine, Inselspital, Bern University Hospital, University of Bern, Bern, Switzerland; 11 Institute of Family Medicine (IMF), University of Fribourg, Fribourg, Switzerland; 12 Children’s Hospital of Eastern Switzerland, Claudiusstrasse 6, 9006 St. Gallen, Switzerland

**Keywords:** SARS-CoV-2, Serology, COVID-19, Children, Household, Antibody

## Abstract

**Background:**

We aimed to determine whether living in a household with children is associated with SARS-CoV-2 seropositivity in adults and investigated interacting factors that may influence this association.

**Methods:**

SARS-CoV-2 serology testing was performed in randomly selected individuals from the general population between end of October 2020 and February 2021 in 11 cantons in Switzerland. Data on sociodemographic and household characteristics, employment status, and health-related history was collected using questionnaires. Multivariable logistic regression was used to examine the association of living with children <18 years of age (number, age group) and SARS-CoV-2 seropositivity. Further, we assessed the influence of reported non-household contacts, employment status, and gender.

**Results:**

Of 2393 working age participants (18–64 years), 413 (17.2%) were seropositive. Our results suggest that living with children and SARS-CoV-2 seropositivity are likely to be associated (unadjusted odds ratio (OR) 1.22, 95% confidence interval [0.98–1.52], adjusted OR 1.25 [0.99–1.58]). A pattern of a positive association was also found for subgroups of children aged 0–11 years (OR 1.21 [0.90–1.60]) and 12–17 years (OR 1.14 [0.78–1.64]). Odds of seropositivity were higher with more children (OR 1.14 per additional child [1.02–1.27]). Men had higher risk of SARS-CoV-2 infection when living with children than women (interaction: OR 1.74 [1.10–2.76]).

**Conclusions:**

In adults from the general population living with children seems associated with SARS-CoV-2 seropositivity. However, child-related infection risk is not the same for every subgroup and depends on factors like gender. Further factors determining child-related infection risk need to be identified and causal links investigated.

**Trial registration:**

https://www.isrctn.com/ISRCTN18181860 .

**Supplementary Information:**

The online version contains supplementary material available at 10.1186/s12916-022-02431-z.

## Background

The role of children and adolescents in the transmission of severe acute respiratory syndrome coronavirus type 2 (SARS-CoV-2) is still not fully understood [1, 2]. Children mostly have mild or no symptoms of coronavirus disease 2019 (COVID-19) if infected by SARS-CoV-2 [3] and long COVID does occur but is much less frequent than in adults [4–6]. While children are rarely seriously affected themselves, the extent to which they can spread the virus and put more vulnerable groups at risk remains debatable.

Household transmission accounts for a substantial number of SARS-CoV-2 infections [7–9]. However, robust data on transmission patterns in household is scarce [10] and evidence on the association between living in a household with children and SARS-CoV-2 seropositivity is inconsistent. Large population-based studies in the UK, Denmark, and France suggest a higher prevalence of SARS-CoV-2 infections for people living in a household with children [11–14]. In contrast, studies among healthcare workers showed an opposite effect [15, 16]. This indicates that there may also be protective effects related to living in a household with children. Whether protective- or risk-increasing effects prevail might depend on the presence of other factors (e.g., time spent at home due to employment status/role as caregiver, behavioral factors such as non-household contacts, adherence to social distancing and hygiene measures). These factors are known to affect both intra- and extra household SARS-CoV-2 transmission risk, but their role is still unknown and thus needs further exploration.

Most studies that have assessed the association of living with children and SARS-CoV-2 infections so far stand out for their large sample sizes. However, many of them are retrospective and based on data from reverse transcriptase polymerase chain reaction (RT-PCR) confirmed infections. As a result, they could overrepresent symptomatic infections, while asymptomatic infections are misclassified, as individuals with subclinical infections are less likely to undergo PCR testing. Furthermore, testing policies have varied throughout the pandemic phases due to reagents availability and testing capacities. Serological studies have been pointed out as reliable tool to understand the full spectrum of symptoms of COVID-19, and the spread of SARS-CoV-2 infections [17, 18]. In combination with a random selection of participants, and pre-defined questionnaires, serological studies allow a more targeted assessment of variables and are less prone to recall- and other information biases.

This study used SARS-CoV-2 serology results and questionnaire data from working age participants (18–64 years) of the *Corona Immunitas* research program in Switzerland [19]. The main objective of this study was to investigate whether living in a household with children is associated with SARS-CoV-2 seropositivity in adults. More specifically we investigated first if SARS-CoV-2 seropositivity in adults is associated with the number of children in the household, and with specific age groups of children and secondly, if this association varies by frequency of contacts outside the household, employment status, and gender.

## Methods

The manuscript has been written following the Consortium for the Standardization of Influenza Seroepidemiology (CONSISE) statement on the Reporting of Seroepidemiologic Studies for Influenza (ROSES-I) [20].

### Context

**Fig. 1:**
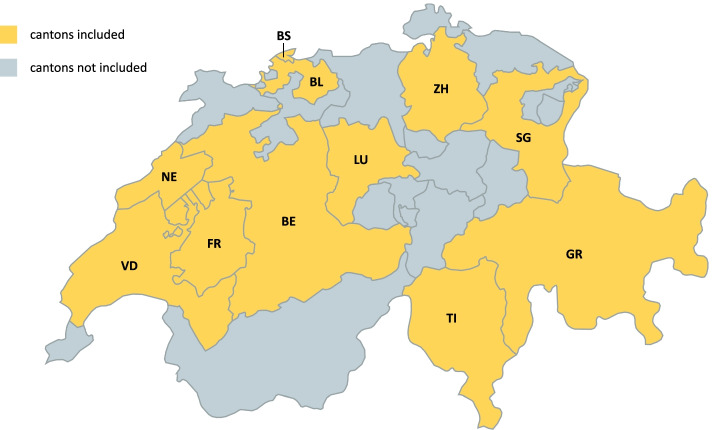
Geographic location of the population-based seroprevalence studies used in this set of analysis. The following cantons are displayed: Basle-City (BS), Basle-Country (BL), Berne (BE), Fribourg (FR), Grisons (GR), Lucerne (LU), Neuchatel (NE), St. Gallen (SG), Ticino (TI), Vaud (VD), and Zurich (ZH)

*Corona Immunitas* is a nationally coordinated research program of seroprevalence studies in Switzerland and is characterized by uniform SARS-CoV-2 antibody tests, standardized questionnaires, and protocols [19]. For this set of analyses, we focused on data collected between the end of October 2020 and February 2021 in the following sites across Switzerland: Basle-City (BS), Basle-Country (BL), Berne (BE), Fribourg (FR), Grisons (GR), Lucerne (LU), Neuchatel (NE), St. Gallen (SG), Ticino (TI), Vaud (VD), and Zurich (ZH). The geographic location of the cantons is displayed in Fig. 1. These cantons comprise a total population of around 5.9 million (roughly 69% of the Swiss population), living in both urban and rural areas, and represent all language regions (German, French, Italian, and Romansh).

**Fig. 2:**
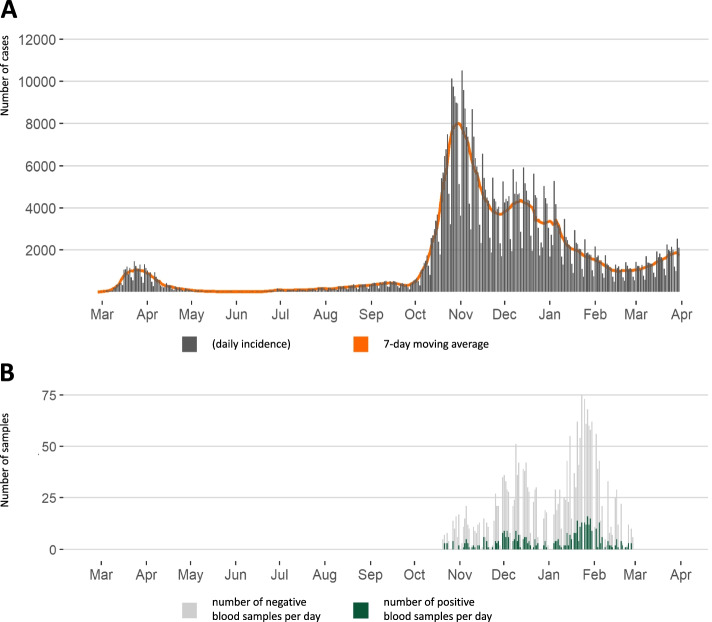
Incidence of SARS-CoV-2 cases in Switzerland and total number of daily samples during the data collection period (March 2020–April 2021). **A** Total number of daily RT-PCR confirmed SARS-CoV-2 cases in Switzerland for the period of March 2020–April 2020 obtained from official statistics [21]. **B** Total number of positive and negative daily blood samples across all study sites (SenASTrIS antibody test result)

A timeline of SARS-CoV-2 incidence in Switzerland is given in Fig. 2A. Switzerland had one of the highest second waves in Europe in autumn 2020. The SARS-CoV-2 vaccination campaign in Switzerland started in most cantons in January 2021, prioritizing the immunization of people at high risk of severe disease and healthcare workers. The serostatus of the study population remained barely affected by SARS-CoV-2 vaccination, as the vaccination campaign initially progressed at low speed, mainly due to limited vaccine supply.

### Study design and participants

Participants were randomly selected from the residential registries of participating cantons provided by the Swiss Federal Statistical Office and invited to participate by postal mail or email. Excluded from this registry are diplomats, persons with a foreign address in the registry, persons in asylum procedure, persons with a short-term residence permit, and elderly people in nursing homes. Invited individuals were asked to schedule an appointment for peripheral venous blood sampling for antibody analysis at one of the study sites. Vulnerable persons (see West et al. [19] for definition) could schedule a home-visit for blood sampling. Study participants did not receive any financial compensation for their participation in the study, except for the travel expenses to the study centre. For this analysis, only working-age participants aged 18–64 years were included. These participants are more likely to be parents or primary caregivers of minors in the same household. Inclusion of older participants might have introduced bias, as this age group is at increased risk for severe COVID-19 and thereby more likely to implement recommended protective measures in their daily lives.

### Serology analysis

The primary outcome variable was the binary SARS-CoV-2 antibody test result. SARS-CoV-2 immunoglobulin A (IgA) and immunoglobulin G (IgG) antibodies become detectable in most cases around 6–15 days after symptom onset (6–10 days for IgA, 11–15 days for IgG) [22]. We analyzed sera extracted from the venous blood using SenASTrIS (Sensitive Anti-SARS-CoV-2 Spike Trimer Immunoglobulin Serological), a Luminex binding assay purposely developed by the Vaud University Hospital (CHUV), the Swiss Federal Institute of Technology in Lausanne (EPFL), and the Swiss Vaccine Center. The assay measures binding of IgG and IgA antibodies to the trimeric SARS-CoV-2 S-protein. The overall test result was counted as positive when either SARS-CoV-2-IgG and/or -IgA signal was above cutoff. Indeterminate test results, meaning a signal just below the predefined cutoff, were retested, but all confirmed “indeterminate” and thus counted as negative. The test has a high specificity (99.7%) and sensitivity (96.6%) and has been validated in samples of the general population as well as specific subgroups of people: individuals with RT-PCR confirmed asymptomatic/paucisymptomatic SARS-CoV-2 infection, individuals with contact to a RT-PCR confirmed SARS-CoV-2 case, and pre-pandemic blood samples of people infected with other viruses (including the human endemic coronaviruses E229, OC43, HKU1, or NL63). A more detailed description of the test and its validation regarding antibody kinetics is available elsewhere [22].

### Questionnaires

Information on the main exposure of interest (living in a household with children), potential confounders, and effect modifying variables was retrieved from the *Corona Immunitas* baseline questionnaires. Participants completed the questionnaires online either at home shortly before the blood sampling appointment or at the testing sites. Information was collected on sociodemographic (age, gender, monthly household income in Swiss francs (CHF), nationality, education) and household characteristics (household size, age, and gender of each household members), employment status (employed/unemployed, working part-time/full-time from home due to SARS-CoV-2 pandemic), health-related behavior, and comorbidities (smoking, diagnosed with cancer, diabetes, hypertension, cardiovascular disease, chronic respiratory disease, or immunocompromised), SARS-CoV-2 compatible symptoms, and tests (previous SARS-CoV-2 RT-PCR or serology tests), as well as information on contacts (number of non-household contacts during the last 7 days for longer than 15 min and less than 1.5 m distance). Household members were considered children if they were younger than 18 years.

### Statistical analysis

Statistical analysis was performed using R statistical software (version 4.0.3, R Foundation for Statistical Computing, Vienna, Austria). The following packages were used: MASS [23], tidyverse [24], knitr [25], tableone [26]. Sociodemographic- and household characteristics, employment status, and underlying health conditions were described stratified by seropositivity. Variables were reported as median and interquartile range (IQR), mean and standard deviation (SD), and frequency and percentage, as appropriate.

**Fig. 3:**
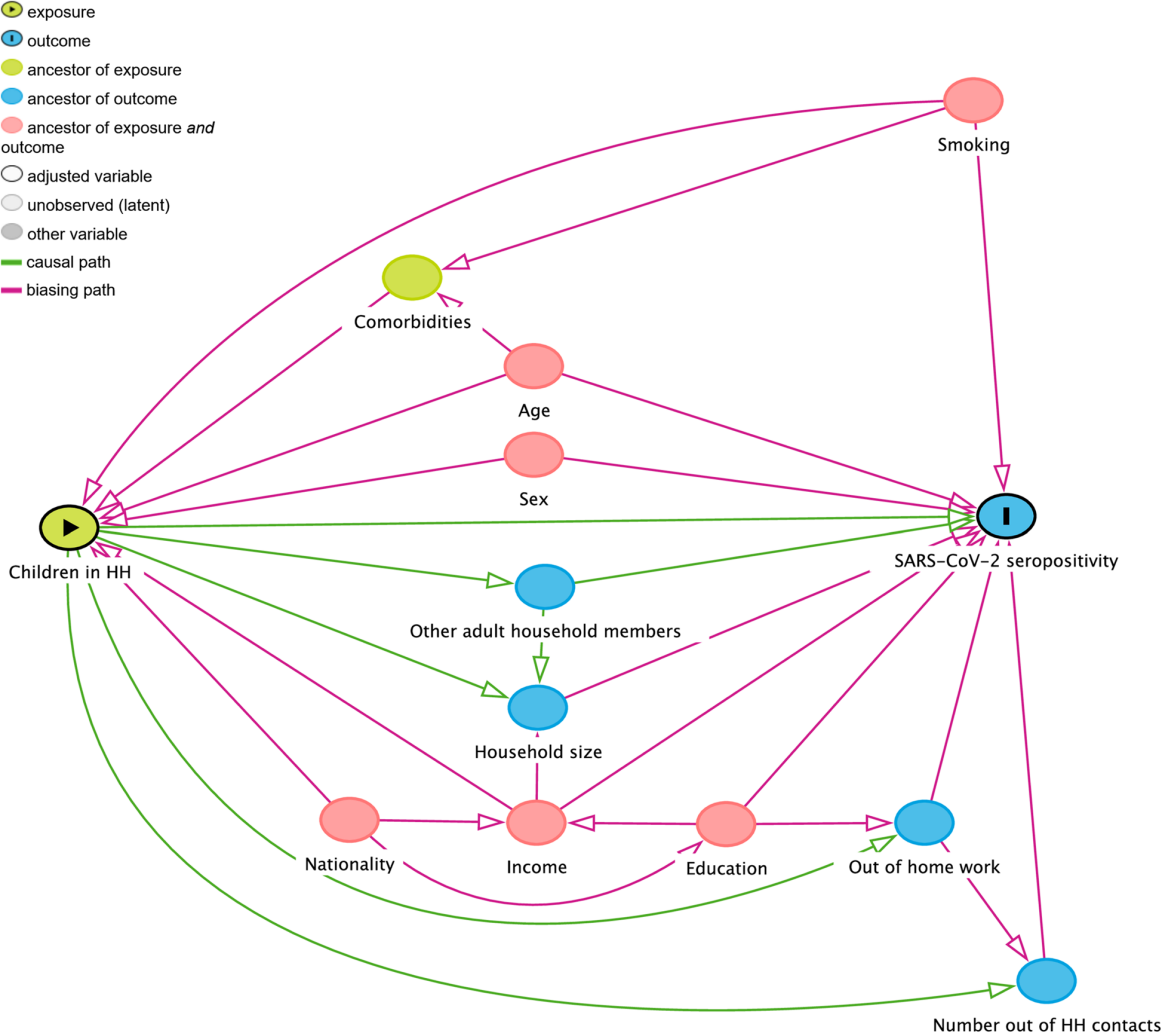
Directed acyclic graph illustrating implicitly assumed causal structure between exposure to children and SARS-CoV-2 seropositivity. Each arrow implies an assumed causal effect from the variable the arrow originates to the variable the arrow is directed at. Variables lying on a directed path between exposure and the outcome of interest (e.g., other adult household members size) do not have to be controlled for. However, variables preceding both exposure and outcome may introduce biases and need to be controlled for. In this case the minimal sufficient adjustment sets for estimating the direct effect of household child exposure on SARS-CoV-2 seropositivity are as follows:• Age, education, income, gender, and smoking. • Age, income, nationality, gender, and smoking. HH household

We used logistic regressions to assess the odds of SARS-CoV-2 seropositivity when living in a a household with children in a complete-case analysis. We ran unadjusted and subsequently adjusted regression models to account for the potential confounding effect of age, gender, income, education, and smoking. Based on what has been described by Forbes et al. [11], confounders were identified using the directed acyclic graph (DAG) approach (see Fig. 3). With DAG, hypothetical relations of variables of interest can be visually summarized. Arrows are used to indicate the direction of the assumed causal effects. Through the application of a standardized set of criteria confounders can be deduced that need to be controlled for to identify the causal effect of interest [27–29].

We then performed the same analysis entering the number of children per household as the main explanatory variable. To determine if the odds of SARS-CoV-2 seropositivity is different when living only with younger children compared to living only with older children, we stratified the analysis by age group (0–11 and 12–17 years, and separately for age 0–5 and 6–11 years). These age groups were formed to reflect school stages and protective measures implemented at schools (age 0–5: pre-school, 6–11: primary school without obligation to wear a mask, 12–17: primary, secondary school, or tertiary education with obligation to wear a mask) and correspond with age groups used in other studies [11, 12, 15, 16]. Participants with children in more than one of the respective age groups were excluded from the analysis on age groups.

We assessed several factors, that potentially modify the risk of SARS-CoV-2 seropositivity related to living with children, as they affect both intra- and extra-household SARS-CoV-2 transmission. We chose employment status (being unemployed or telework versus working outside of home), presence of non-household contacts (approximated by number of reported non-household contacts within the previous seven days), and gender as variables to be assessed for their interacting effects. Both, people not working at home and people with frequent contacts outside of the household contact might have relatively higher risk of becoming infected outside the household [30, 31], while their risk for intra household transmission is lower due to a shorter exposure time. Male gender has been shown to be associated with both a higher intra- and extra household transmission risk [32]. At the same time women, in general and throughout the SARS-CoV-2 pandemic in particular, often spent more time at home in direct contact with children [33]. Each potential effect modifying variable was assessed in a separate multivariable logistic regression model (the same model as used for previously described analysis adjusting for confounders), assessing both the main and interaction effect of the individual variable as well as the interaction term (effect modifying variable × ≥ 1 child in household).

### Sensitivity analysis

Several sensitivity analyses were performed to assess the consistency of our results. For the main analysis, all participants, irrespective whether they live alone or in a multi-person household were included. The majority of SARS-CoV-2 transmission occurs within households [7–9]. For people living alone, this risk is absent. Thus, including individuals living in single-person households might skew the results of people living in a household without children. We therefore reran our logistic regression models including only individuals with at least one other household member.

Although vaccination campaigns in Switzerland started at the end of December 2020, having received at least one dose of a SARS-CoV-2 vaccine was no exclusion criteria for the main analysis. As some of the participants might already have been vaccinated by the time the blood sample was taken, we reran our analysis excluding people that have reported to have received at least on vaccine dose as part of our sensitivity analysis.

When using the DAG-approach to identify confounders, the direction of the relationship of having children and smoking was not distinct. Our initial DAG assumed that there is a self-selection of healthier people becoming parents [34], turning smoking into a confounder that requires adjustment. However, having children also leads to parents living healthier lives [35, 36]. In this case, the relationship between smoking and living with children is inverse, and smoking, acting as a mediator in this case, would not need to be controlled for. Thus, as part of our sensitivity analysis, we also assessed the association of living with children and SARS-CoV-2 seropositivity in a multivariable logistic regression model without smoking as a confounder.

## Results

Overall participation rate across all study sites was 18.8% (range 11.7 to 25.5%). Antibody test results were available for 4273 participants, of which 2393 were 18–64 years old. We excluded 1838 participant aged 65 years and older, of which only 15 reported to live in a household with children, and 42 participants younger than 18 years. Of 2393, 413 participants (17.3%) were seropositive according to the Luminex test (68 only IgA-positive, 83 only IgG-positive, 262 both IgA and IgG-positive). Fifteen participants (0.8%) were seronegative but reported a positive SARS-CoV-2 PCR-test in the questionnaire. Ten participants (0.4%) reported a SARS-CoV-2-related hospital stay. Of those with known vaccination status, 13 (0.5%) participants reported to have received at least one dose of SARS-CoV-2 vaccination, of which 5 were seropositive. The progress of SARS-CoV-2 serology testing and number of daily positive and negative blood samples is displayed in Fig. 2B.

Characteristics of the study population are shown in Table 1. In total, 827 participants (34.8%) reported to live in a household with at least one child (range: 1–7 children per household, mean 1·74 (standard deviation: 0·74) per participant’s household). The median age of children was 9 years (interquartile range (IQR) = 4–13 years). Of all participants living with children, 459 (55.5%) lived only with children aged 0–11 years, 244 (29.5%) lived only with children aged 12–17 years and 124 (15.0%) lived with children of both age groups.

**Table 1:** Characteristics of the study population stratified by binary SenASTrIS antibody test result

Variable	Negative ^**a**^ (***n*** = 1980)	Positive ^**a)**^ (***n*** = 413)
**SARS-CoV-2 symptoms and tests**		
Prior PCR-confirmed SARS-CoV-2 infection	15 (0.8)	128 (31.2)
Symptoms ^b^		
None	816 (42.2)	93 (22.8)
< 3	323 (16.7)	51 (12.5)
≥ 3	795 (41.1)	264 (64.7)
**Primary exposure**		
Number of children in household ^c^	0.59 (±0.92)	0.70 (±1.01)
Children in household		
None	1296 (66.0)	252 (61.3)
Any child 0–5 years	276 (14.1)	64 (15.6)
Any child 6–11 years	279 (14.2)	71 (17.3)
Any child 12–17 years	296 (15.1)	72 (17.5)
Child with PCR-confirmed infection	17 (0.9)	13 (3.2)
**Sociodemographic characteristics**		
Age ^c)^	45·04 (±12.3)	43·97 (±11.9)
Male gender	917 (46.4)	202 (49.0)
Household monthly income (CHF) ^d^		
< 6000	521 (28.0)	113 (29.4)
6000–<12,000	908 (48.8)	176 (45.8)
12,000–<18,000	314 (16.9)	65 (16.9)
≥ 18,000	116 (6.2)	30 (7.8)
Swiss nationality	1681 (85.4)	346 (84.2)
Highest education		
Primary	83 (4.2)	17 (4.1)
Secondary	878 (44.7)	162 (39.5)
Tertiary	1002 (51.0)	231 (56.3)
**Other household characteristics**		
Number of other people in the household ^c^	1.86 (±1.29)	1.99 (±1.34)
≥ 1 other adult in household	1678 (85.4)	356 (86.6)
Number of other adults in the household ^c^	1.25 (±0.91)	1.23 (±0.92)
**Employment status and other exposure**		
Being unemployed *or* working full-time from home	879 (47.1)	141 (36.2)
≥ 1 contact outside the household during the previous 7 days	1608 (83.2)	335 (83.8)
Number of contacts outside the household during previous 7 days ^c^	8·58 (±27.2)	9·80 (±21.8)
Notification by SwissCOVID App about contact tested positive	51 (5.4)	18 (10.4)
*Health-related history*		
Any chronic conditions ^e^	338 (17.2)	74 (18.0)
Cancer	16 (0.8)	2 (0.5)
Diabetes	30 (1.5)	11 (2.7)
Immunocompromised	51 (2.6)	10 (2.4)
Hypertension	184 (9.4)	32 (7.8)
Cardiovascular disease	35 (1.8)	11 (2.7)
Chronic respiratory disease	106 (5.4)	26 (6.3)
Smoking tobacco products	481 (24.4)	98 (23.8)

If not indicated otherwise, data are presented as count (%)

^a^Indeterminate results (*n* = 44) were all confirmed “indeterminate” and thus considered negative

^b^Any of the following: fever (subjective), fever (higher 38°C), cough, rhinorrhoea, sneezing, sore throat, shortness of breath, trouble breathing, headache, myalgia, chest pain, fatigue, loss of appetite, nausea, diarrhoea, upset stomach, and anosmia for at least 3 days

^c^Presented as mean with (± standard deviation)

^d^In 2020 on average 1 CHF was equal to 0.93 €, and

^e^Any of the following: cancer, diabetes, immunocompromised, hypertension, cardiovascular disease, and chronic respiratory disease

### Association of living with children and SARS-CoV-2 seropositivity

Results of the logistic regression analysis are reported in Table 2. In both unadjusted and adjusted analyses (see unadjusted analysis and Model 1, Table 2), a trend of higher odds of SARS-CoV-2 seropositivity was observed in adults when living in a household with children with similar odds ratios in both models.

**Table 2:** Logistic regression analysis assessing the association of living with children and SARS-CoV-2 seropositivity

Exposure	Odds ratio (95% CI)
**Model 0: Any child in household, unadjusted analysis** ***(2375 complete cases)***	
No child in household	[Reference]
≥ 1 child in household	1.22 (0.98–1.52)
**Model 1: Any child in household, adjusted analysis** ***(2232 complete cases)***	
No child in household	[Reference]
≥ 1 child in household	1.25 (0.99–1.58)
**Model 2: Age of children in household** ***(2232 complete cases, 2114 included*** ^***a)***^***)***	
No children in household	[Reference]
Only children 0–11 years	1.21 (0.90–1·60)
Only children 12–17 years	1.14 (0.78–1·64)
**Model 3: Number of children in household** ***(2232 complete cases)***	
Number of children in household (per child)	1.14 (1.02–1.27)
**Model 4: Interaction of living with children and out of household contacts** ***(2197 complete cases)***	
No child in household	[Reference]
≥ 1 child in household	1.21 (0.69–2.10)
No non-household contact in previous 7 days	[Reference]
≥ 1 non-household contact in previous 7 days	0.97 (0.66–1.47)
Interaction	1.06 (0.58–1.96)
**Model 5: Interaction of living with children and employment status** ***(2122 complete cases)***	
No child in household	[Reference]
≥ 1 child in household	1.44 (1.06–1.93)
Working outside home	[Reference]
Being unemployed *or* working full-time from home	0.69 (0.51–0.94)
Interaction	0.67 (0.41–1.09)
**Model 6: Interaction of living with children and gender of participant** ***(2232 complete cases)***	
No child in household	[Reference]
≥ 1 child in household	0.95 (0.68–1.32)
Female gender	[Reference]
Male gender	0.87 (0.65–1.16)
Interaction	1.74 (1.10–2.76)

Models 1–6 are adjusted for age, gender, income, education, and smoking

^a^88 cases were excluded as they included children of both age groups (living with children 0–11 years and 12–17 years)

Odds ratios for SARS-CoV-2 seropositivity when living with children were similar for the subgroup of children aged 0–11 and 12–17 years. However, associations were not as clear and confidence intervals were wider (see Model 2, Table 2). This was also observed after subdividing participants living with 0–11-year-old children into those living with 0–5- or 6–11-year-old children (for children 0–5 years: odds ratio (OR) 1.24 and 95% confidence interval [0.84–1.80], for children 6–11 years: OR 1.39 [0.88–2.14]).

We observed a positive association between SARS-CoV-2 seropositivity and number of children in the household (see Model 3, Table 2). A strong trend of higher odds of SARS-CoV-2 seropositivity with every additional child in the household remained after we accounted only for participants with at least two children in the household.

### Effect modification by employment status, number of non-household contacts, and gender

Odds of SARS-CoV-2 seropositivity were not higher for individuals with non-household contacts within the previous seven days (see Model 4, Table 2). Being unemployed or working full-time from home was associated with lower odds of seropositivity (see Model 5, Table 2). Although some interacting effect of work situation might be possible, interaction terms in both models showed no clear picture that either variable altered odds of SARS-CoV-2 seropositivity in people living with children (see interaction terms Models 4 and 5, Table 2). This was different for gender, with male participants who live in a household with children having substantially higher odds of seropositivity compared to women (see interaction term Model 6, Table 2). Gender as individual variable was not associated with seropositivity in neither of the models.

### Sensitivity analysis

Both the exclusion of participants living in a single-person household (unadjusted OR 1.20 [0.95–1.51], adjusted OR (1.23 [0.97–1.57]) and the exclusion of people having received at least one SARS-CoV-2 vaccine (unadjusted OR 1.21 [0.97–1.50], adjusted OR 1.23 [0.97–1.55]) did not substantially alter the result compared to the initial analysis.

Also, no substantial alteration of results was observed after removing smoking as a confounder in the multivariable analysis (OR 1.25 [0.99–1.58]).

## Discussion

In this cross-sectional study, we analyzed the association between SARS-CoV-2 seropositivity and living in a household with children, based on questionnaire and serology data from population-based SARS-CoV-2 seroprevalence studies in 11 cantons in Switzerland. Our results suggest that for the general population overall odds of SARS-CoV-2 seropositivity may be higher when a household is shared with at least one child of any age. However, despite this association in the general population, risk of SARS-CoV-2 infection related to living in a household with children might vary between subgroups. Indeed, interaction analyses indicated higher odds of SARS-CoV-2 seropositivity when living in a household with children for men than for women.

Although the precision (described by the confidence interval) of the overall analysis limits our ability to make definite general statements, the finding of a positive association of living with children and SARS-CoV-2 seropositivity in our sample of general adult population in Switzerland is consistent with other population-based SARS-CoV-2 studies [11–14]. Results of the multivariable analysis in our study were very similar to what has been observed by Carrat et al. based on seroprevalence data in France, where a comparable population-based SARS-CoV-2 antibody testing approach has been followed [13]. A positive association of living with children and SARS-CoV-2 infection was also reported in population-based studies in Denmark [12] and during the second wave in the UK [11], which have calculated hazard ratios based on data from PCR-confirmed infections. While taken these results together, all studies agree that for the general population an association is very likely, our study in line with the other studies indicates that the strength of this association is only weak to moderate. Neither adjusted odd ratios in our study (OR 1.25 [0.99–1.59]) and reported by Carrat et al. from France (OR 1.3 [1.11–1.53]) nor hazard ratios reported by Husby et al. from Denmark (hazard ratio 1.05 [1.02–1.09] and by Forbes et al. from the second wave in the UK (hazard ratio 1.06 [1.05–1.08] and 1.22 [1.20–1.24] for living with children aged 0–11 and 12–17, respectively) indicates a strong increase in risk of SARS-CoV-2 when a household is shared with children [11–13].

Next to our observations on the association of living with children and SARS-CoV-2 infection in general, we observed that the odds of SARS-CoV-2 seropositivity increases with every additional child in the household. A similar finding has been made by Husby et al. based on PCR-data in Denmark, which has shown a positive trend of higher hazard ratios for SARS-CoV-2 infection when living in a household with one, two, and three or more children, respectively [12]. Odds ratios were similar for all children’s age groups. Results were comparable to the observations made in the overall analysis, although confidence intervals were wider (likely due to the lower number of cases in this subanalysis). Therefore, our results do not indicate that living with children of a particular age group has a stronger association with seropositivity than living with children of other age groups. Differences in number of non-household contacts have been proposed as a potential behavioral reason for an altered SARS-CoV-2 infection risk, when living with children. For example, Husby et al. suggested that living with children might lead to more non-household contacts as caregivers accompany their children on playdates [12] while another study suggests people living with children have less non-household contacts, because they are more likely to stay at home and spend time as a family [15]. In our study, the odds of SARS-CoV-2 seropositivity were not higher for people with at least one non-household contact reported within the previous seven days. Thus, differences in contact patterns likely do not explain an altered SARS-CoV-2 infection risk related to living in a household with children. In line with other studies [14, 31], being unemployed or working from home was inversely associated with seropositivity, which is likely explained by the substantially lower work and commute-related risk of SARS-CoV-2 transmission. Interaction analyses for both non-household contacts and employment status showed no clear picture of an effect modification. Consistent with findings from other studies, SARS-CoV-2 seropositivity related to living with children was dependent on gender [11, 12]. While in our analysis SARS-CoV-2 seropositivity was not associated with gender itself, male participants had substantially higher rates of SARS-CoV-2 seropositivity if they lived in a household with children.

As antibody tests cannot identify the exact timepoint of infection and participants’ household members were not tested, we were unable to define transmission routes or determine the extent to which living with children and SARS-CoV-2 infections in adults are causally related. Children being able to get infected and transmit the virus to others could impose a direct risk to adults living in the same household. At the same time, there are other individual characteristics and behavioral factors that are associated with sharing a household with children, which could be of importance regarding SARS-CoV-2 infection risk.

There is some evidence indicating that children could increase the risk of a SARS-CoV-2 infection in household members by being a direct source of infection. PCR-based studies have shown a higher secondary attack rate of paediatric compared to adult index cases in households [37, 38]. With milder clinical manifestations [39–41], SARS-CoV-2 infections in children tend to remain undiagnosed [42] and unnoticed SARS-CoV-2 infections in children could keep households from implementing necessary isolation measures to interrupt transmission chains. Further, young children cannot be isolated from their caregivers when being sick [43], and social distancing and hygiene measures are more difficult to implement. Also, restrictions in extra-curricular activities in early 2021 in Switzerland were limited only for children older than 12 years [2] and apart from a relatively short period during the first wave in March–April 2020, schools in Switzerland have remained open. School-aged children and adolescents have shown to be more mobile and tend to have more close contacts with individuals outside the household [44]. As such, the relative risk of children to acquire a SARS-CoV-2 infection outside the household might have been substantially higher in comparison to adults.

On the contrary, several studies have shown that children only account for a very small proportion of index cases within household clusters [45–47] and infectivity is lower when index cases are asymptomatic [9, 37, 48], as it is frequently the case in children. Our results provide no evidence that the odds of SARS-CoV-2 seropositivity are substantially increased in individuals spending more time at home with contact to children (e.g., people being unemployed or performing work from home, people having no non-household contacts). Furthermore, our results and other studies [15, 16] show that although there is an association in the general population not everybody living with children is at increased risk of SARS-CoV-2 infection and for certain groups even the opposite is the case (i.e., odds of SARS-CoV-2 infections are lower when living with children). In our study, SARS-CoV-2 seropositivity for men living with children was substantially increased. A negative association between living with children and SARS-CoV-2 seropositivity was also observed among healthcare workers in Scotland [15] and Switzerland [16].

This suggests that risk of SARS-CoV-2 infection related to living with children is not only determined by the child’s infectivity, but other characteristics and behavioural factors do play a role, of which some can also be protective. One of those protective effects might be related to seasonally spreading human endemic coronaviruses, due to which children might possess some cross-immunity to SARS-CoV-2 [49, 50], and more frequent co-infections with other viruses could interfere with the replication of SARS-CoV-2 [49]. Individuals spending a lot of time at home taking care of children could profit of such cross-immunity and other competing infections, due to increased exposure. This effect could be particularly prominent in women who, already before the pandemic, more often spent time at home taking care of children [33]. Living with children further leads to more part-time work, as more time is allocated to childcare. People working in professions with high risk of SARS-CoV-2 transmission such as healthcare [51, 52] could thereby profit from living with children, as they spend less time at work being exposed to SARS-CoV-2. At the same time, in healthcare workers, social distancing and hygiene measures might have been better implemented, that have shown to significantly reduce transmission risk from a child, if infected [53]. Although we were unable to assess these factors related to working in healthcare jobs in our study, this could to some extent explain the discrepancies of our findings in comparison with studies conducted among healthcare workers [15, 16].

With our study design using retrospectively reported symptoms, we were unable to define if severity of COVID-19 is different for people living with children versus people living without. Number of SARS-CoV-2-related hospital admissions was low in our population-based cohort (total number: 10, of which 3 living with children). Thus, in contrast to previous studies [11, 12, 15], we were unable to use this variable as a proxy to assess severity of COVID-19. An additional analysis on self-reported symptoms in participants revealed that most symptoms occur with similar frequencies in seropositive individuals with children compared to seropositive individuals without children (see additional file 1: Table S1). For seronegative individuals, symptoms were generally more frequent in people living with children compared to people living without, indicating that people living in a household with children might get more often sick, but this is likely not related to SARS-CoV-2.

Despite its strength of being a large nation-wide population-based study using a highly sensitive and specific SARS-CoV-2 antibody test, this study has some limitations. Overall participation rate was moderate and non-random willingness to participate in the survey is possible (e.g., higher participation of more highly educated individuals, individuals who experienced symptoms suggestive of COVID-19, individuals with a confirmed SARS-CoV-2 infection, or individuals who were exposed or believed themselves to be exposed to SARS-CoV-2). However, we have no reason to believe that non-random willingness to participate in the study has been substantially different among people living with compared to people living without children. Although some selection bias cannot be ruled out, we expect the effect, if any, to be of very small influence on the overall results. Due to the limited number of participants, some associations could not be estimated precisely. Especially results of the analysis of subgroups as well as interaction analysis should thus be interpreted with caution. Participants’ characteristics were furthermore self-reported, and some variables could be only assessed as approximations (see number of non-household contacts). Accuracy on some variables might be limited and, in some cases, recall bias could have occurred. Despite the high sensitivity and specificity of the antibody test used, some misclassification is possible and effect sizes may be biased towards the null and thus some associations could have been missed. Especially in case of SARS-CoV-2 infections that date back longer, SARS-CoV-2-specific antibodies might have waned. However, as we expect this to have happened irrespective of living with children, overall results are likely not substantially affected. In general, with serology results, we were unable to determine the timepoint when infection occurred. Infection risk related to living with children could have been different with different transmission dynamics at different stages of the SARS-CoV-2 pandemic in Switzerland. Our study most accurately depicts the pandemic situation during a time of high community transmission during the second wave in Switzerland. Given that SARS-CoV-2 antibodies take around 6–15 days to become detectable [22], very few samples taken as part of our study (those that were collected in October 2020, when community transmission was just starting to rise in Switzerland), would not represent second-wave transmission dynamics. Transmissions within households with the delta and omicron variant of SARS-CoV-2 appears to be further increased [54, 55]. However, these strains did not become prevalent in Switzerland until May 2021 (delta variant) and November 2021 (omicron variant), respectively [21], later than our data collection. Next to variation in containment measures, intra household transmission could have been lower at later stages of the pandemic, because with increasing number of immune household members (due to previous infection or vaccination) also non-immune household members are protected [56].

## Conclusions

SARS-CoV-2 seropositivity in the general population may be higher when living in a household with children, but increased SARS-CoV-2 infection risk, if any, was not found to be particularly strong. Moreover, the risk is not the same for every subgroup and might be higher for men compared to women. Although it is impossible to establish direct causal links with our study design, it is likely that several risk increasing and protective effects related to living with children play a role. Further research is needed to understand the mechanisms for these effects. This could eventually allow for more targeted statements about risks to specific subgroups.

## Supplementary Information

**Additional file 1: Table S1.** Symptoms and SARS-CoV-2 PCR-tests stratified by living in a household with children and binary SenASTrIS antibody test result.

## Data Availability

The datasets generated and/or analyzed during the current study are currently not publicly available as data relating to the *Corona Immunitas* research program is still being collected. Deidentified participant data might be available on reasonable request by email to the corresponding author at later stages of the study.

## Abbreviations

SARS-CoV-2: Severe acute respiratory syndrome coronavirus type 2
COVID-19: Coronavirus disease 2019
RT-PCR: Reverse transcriptase polymerase chain reaction
IgA: Immunoglobulin A
IgG: Immunoglobulin G
SenASTrIS: Sensitive Anti-SARS-CoV-2 Spike Trimer Immunoglobulin Serological
IQR: Interquartile range
SD: Standard deviation
DAG: Directed acyclic graph
OR: Odds ratio
