# Supplementary material for: Is living in a household with children associated with SARS-CoV-2 seropositivity in adults? Results from the Swiss national seroprevalence study Corona Immunitas
Source: BMC Med. 2022 Jun 20;20:233. doi: 10.1186/s12916-022-02431-z (PMC9207841; doi:10.1186/s12916-022-02431-z)
Supplement: Supplementary file 1 — Additional file 1: Table S1. Symptoms and SARS-CoV-2 PCR-tests stratified by living in a household with children and binary SenASTrIS antibody test result. [file 12916_2022_2431_MOESM1_ESM.docx]

# SUPPLEMENTARY MATERIAL

**Table S1:** Symptoms and SARS-CoV-2 PCR-tests stratified by living in a household with children and binary SenASTrIS antibody test result.

|  | **Without children in household** | | **With children in household** | |
| --- | --- | --- | --- | --- |
| **SARS-CoV-2 SEROLOGY** | **NEGATIVE**  **(n = 1296)** | **POSITIVE**  **(n = 252)** | **NEGATIVE**  **(n = 668)** | **POSITIVE**  **(n = 159)** |
| **SARS-CoV-2 PCR** |  |  |  |  |
| Prior PCR-test | 348 (26·9) | 108 (42·9) | 216 (32·4) | 96 (60·4) |
| Positive PCR-test | 8 (0·6) | 65 (25·9) | 7 (1·1) | 63 (39·6) |
| **Symptoms** |  |  |  |  |
| ≥ 1 symptom | 772 (60·2) | 199 (79·3) | 480 (72·5) | 138 (87·9) |
| Number of symptoms ^a)^ | 2·76 (±3·49) | 4·90 (±4·29) | 3·72 (±3·82) | 5·52 (±4·11) |
| Fever (subjective) | 243 (18·8) | 95 (37·7) | 170 (25·5) | 68 (43·0) |
| Fever (higher 38° C) | 109 (8·4) | 63 (25·0) | 83 (12·5) | 40 (25·2) |
| Cough | 256 (19·9) | 95 (37·7) | 153 (23·0) | 64 (40·3) |
| Rhinorrhoea | 477 (36·9) | 124 (49·4) | 320 (48·2) | 97 (61·4) |
| Sneezing | 393 (30·5) | 103 (41·5) | 268 (40·4) | 73 (46·5) |
| Sore throat | 359 (27·9) | 89 (35·7) | 251 (37·9) | 69 (43·9) |
| Shortness of breath | 101 (7·9) | 54 (21·6) | 67 (10·1) | 38 (24·4) |
| Trouble breathing | 67 (5·2) | 34 (13·8) | 48 (7·3) | 19 (12·2) |
| Headache | 378 (29·3) | 107 (43·0) | 254 (38·2) | 79 (49·7) |
| Myalgia | 252 (19·7) | 116 (46·2) | 173 (26·2) | 70 (44·6) |
| Chest pain | 110 (8·5) | 41 (16·5) | 69 (10·4) | 26 (16·7) |
| Fatigue | 390 (30·2) | 130 (51·8) | 286 (43·0) | 88 (55·7) |
| Loss of appetite | 94 (7·3) | 37 (15·0) | 60 (9·1) | 22 (14·3) |
| Nausea | 79 (6·1) | 26 (10·4) | 61 (9·2) | 20 (12·8) |
| Diarrhoea | 154 (12·0) | 49 (19·7) | 121 (18·3) | 29 (18·5) |
| Upset stomach | 122 (9·5) | 37 (15·0) | 90 (13·6) | 25 (16·1) |
| Anosmia | 36 (2·8) | 75 (29·9) | 26 (3·9) | 54 (34·6) |

If not indicated otherwise, data are presented as count (%). a) presented as mean with (± standard deviation)
